# Analgesic, Anti-Inflammatory, and Chondroprotective Activities of *Siraitia grosvenorii* Residual Extract

**DOI:** 10.3390/ijms25084268

**Published:** 2024-04-12

**Authors:** Yun-Mi Lee, Dong-Seon Kim

**Affiliations:** KM Science Research Division, Korea Institute of Oriental Medicine, Daejeon 34054, Republic of Korea; candykong@kiom.re.kr

**Keywords:** *Siraitia grosvenorii* residue extract, anti-inflammation, interleukin-1β, NF-κB signalling pathway, osteoarthritis, chondroprotection

## Abstract

Inflammation is crucial to osteoarthritis (OA) pathogenesis. The aim of this study was to evaluate *Siraitia grosvenorii* residue extract (NHGRE) obtained by extracting *S. grosvenorii* fruits with water as a potential food supplement for treating arthritis based on its analgesic, anti-inflammatory, and chondroprotective effects and the remaining residue with 70% ethanol. We observed the analgesic activity of NHGRE based on the acetic acid-induced writhing response in mice, examined its anti-inflammatory efficacy against carrageenan-induced paw oedema in mice, and investigated its effect on inflammatory cytokine expression in interleukin (IL)-1β-induced SW1353 cells. Furthermore, we determined its effects on cartilage protection in interleukin-1β (IL-1β)-treated SW1353 cells. NHGRE at 200 mg/kg significantly reduced the acetic acid-induced writhing response and prevented oedema formation in the carrageenan-induced paw oedema model. In IL-1β-induced SW1353 cells, NHGRE at 400 µg/mL reduced the expression of inflammation mediators such as tumour necrosis factor (TNF)-α (55.3%), IL-6 (35.4%), and prostaglandin E2 (PGE2) (36.9%) and down-regulated the expression of matrix metalloproteinase (MMP)-1 (38.6%), MMP-3 (29.3%), and MMP-13 (44.8%). Additionally, it restored degraded collagen II levels in chondrocytes. NHGRE plays a protective role in chondrocytes by regulating Nuclear factor kappa B (NF-κB) activation. Overall, NHGRE may be a useful therapeutic agent for OA by controlling pain, oedema formation, and inflammation-related mechanisms.

## 1. Introduction

Inflammation is a complex biological response that involves the activation of enzymes, release of various chemical mediators, migration of cells, release of fluids, and damage and repair of tissues [1]. Inflammation is a common pathogenesis of chronic diseases, including arthritis, diabetes, cancer, and cardiovascular and intestinal diseases [2]. The symptoms of inflammation in osteoarthritis (OA) include redness, joint oedema, stiffness, and joint function loss. The long-term pain experienced in OA is primarily caused by chronic inflammation [3]. The expression of pro-inflammatory factors and matrix degrading enzymes is up-regulated in OA chondrocytes [4]. Previous studies have shown that several inflammatory mediators including interleukin (IL)-1β and IL-6 are involved in OA progression [5]. In particular, IL-1β plays an essential role in OA progression by exacerbating inflammatory responses and the expression of over-induction-associated catabolic enzymes and inflammatory factors such as matrix metalloproteinases (MMPs), aggrecanases, cyclooxygenase (COX)-2, inducible nitric oxide synthase (iNOS), prostaglandin E2 (PGE2), and a disintegrin and metalloproteinase with thrombospondin motifs (ADMATs). This leads to extracellular matrix (ECM) degradation in chondrocytes [6,7].

*Siraitia grosvenorii*, which is known as ‘monk fruit’ or ‘*nahangwa* (NHG) (in Korea)’, is a perennial vine belonging to the Cucurbitaceae family. It is used in traditional Chinese medicine to treat throat, cough, asthma, and intestinal ailments [8,9]. Pharmacological studies have shown that *S. grosvenorii* exhibits antihyperglycaemic, antitumour, immune-enhancing, antioxidant, and protective effects in lung injury and intestinal damage [10,11,12,13,14,15]. *S. grosvenorii* fruit extract is naturally low in calories and contains sweet cucurbitane glycosides. Therefore, for commercial use as a sugar substitute sweetener, *S. grosvenorii* fruits are extracted with water, and the residue (NHGR) is usually discarded [9]. NHGR is a raw material available at a low cost and contains several effective compounds that are not extracted with water, and our previous study showed that this residue exerts anti-inflammatory effects in vitro and anti-osteoarthritis effects in vivo [16]. Hence, in the present study, we aimed to elucidate the antinociceptive and anti-inflammatory effects of *S. grosvenorii* residue extract (NHGRE) in an animal model and its chondroprotective activities in a chondrocyte model as these aspects of the extract have not been described yet. For this purpose, we examined NHGRE as a potential food supplement for treating arthritis based on its analgesic, anti-inflammatory, and chondroprotective effects.

## 2. Results

### 2.1. NHGRE Down-Regulates COX and 5-LOX Activities

NHGRE down-regulated COX-1 and COX-2 enzyme activities in a dose-dependent manner, and its IC_50_ was found to be 62.00 and 82.33 μg/mL, respectively. Additionally, 5-lipoxygenase (5-LOX) activity was suppressed by NHGRE, with an IC_50_ of 270.95 μg/mL (Table 1).

### 2.2. Effect of NHGRE on Acetic Acid-Induced Writhing Response

**Figure 1:**
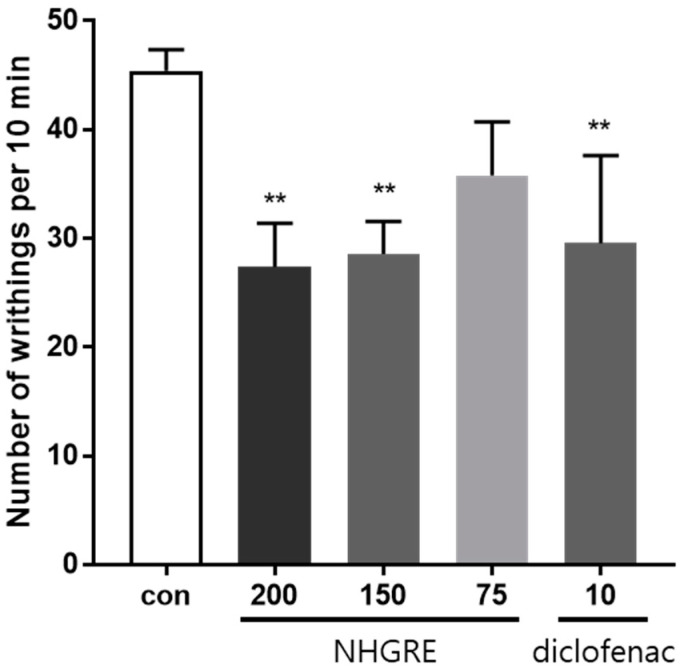
Inhibitory effect of NHGRE and diclofenac on abdominal constriction in mice treated with acetic acid. Values are expressed as mean ± SD. ** *p* < 0.01 vs. Con. *n* = 5.

As shown in Figure 1, treatment with NHGRE (75, 150, and 200 mg/kg) and diclofenac (10 mg/kg) significantly reduced acetic acid-induced abdominal writhing by 21.2%, 37.0%, 39.7%, and 34.8%, respectively, compared to the control. These results demonstrate that NHGRE has an analgesic effect on the acetic acid-induced writhing response.

### 2.3. Effect of NHGRE on Carrageenan-Induced Paw Oedema

**Figure 2:**
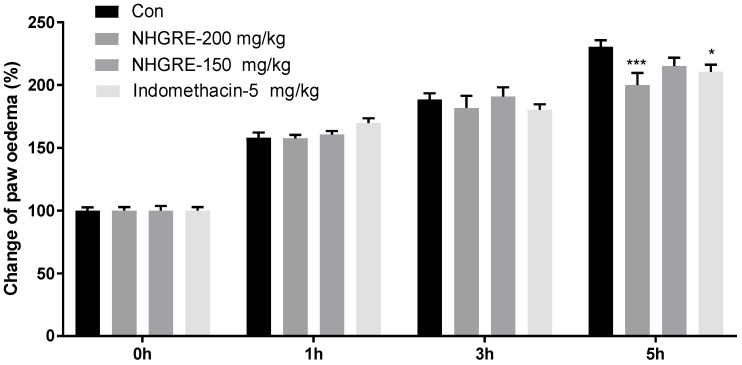
Inhibitory effect of NHGRE and indomethacin on carrageenan-induced oedema in hind paw in rats. Volume of paw oedema was evaluated at 0, 1, 3, and 5 h after carrageenan injection. Values are expressed as mean ± SD. * *p* < 0.05 and *** *p* < 0.001 vs. Con. *n* = 5.

As shown in Figure 2, carrageenan injection increased the paw thickness and degree of swelling in a time-dependent manner, reaching the maximum percentage change from baseline values at 5 h. Pretreatment with NHGRE (200 mg/kg) and indomethacin reduced paw oedema by 23.3% and 15.2%, respectively, compared to the difference in oedema between 0 and 5 h in vehicle control mice.

### 2.4. Effect of NHGRE on TNF-α, IL-6, and PGE2 Production in IL-1β-Treated SW1353 Cells

**Figure 3:**
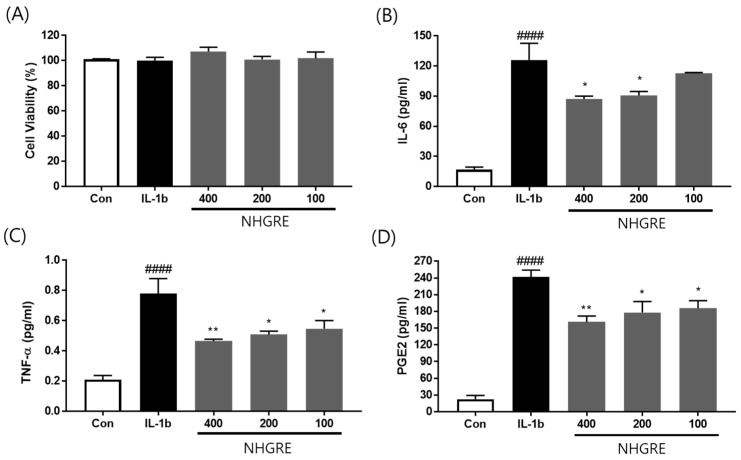
The reduction in inflammatory cytokine expression by NHGRE in IL-1β-stimulated SW1353 chondrocytes. (**A**) The cell viability assay following NHGRE treatment. The concentrations of IL-6 (**B**), TNF-α (**C**), and PGE2 (**D**) were measured in the culture media using a commercial enzyme-linked immunosorbent assay (ELISA) kit. Each value represents the mean ± SD. ^####^ *p* < 0.0001 compared to the untreated control. * *p* < 0.05 and ** *p* < 0.01 compared to the IL-1β-stimulated group. *n* = 3.

As shown in Figure 3B–D, the expression of TNF-α, IL-6, and PGE2 was markedly elevated by IL-1β treatment in the SW1353 cell culture medium. In contrast, IL-6 expression was significantly suppressed, by 12.0%, 32.2%, and 35.4%, respectively, after treatment with 100, 200, and 400 μg/mL NHGRE (Figure 3B). TNF-α expression decreased by 40.9%, 47.7%, and 55.3%, respectively, in cells treated with NHGRE at 100, 200, and 400 μg/mL (Figure 3C). NHGRE treatment at 100, 200, and 400 μg/mL significantly decreased PGE2 production in IL-1β-stimulated cells by 25.7%, 29.1%, and 36.9%, respectively (Figure 3D).

### 2.5. Effects of NHGRE on MMP Expression in IL-1β-Stimulated SW1353 Cells

**Figure 4:**
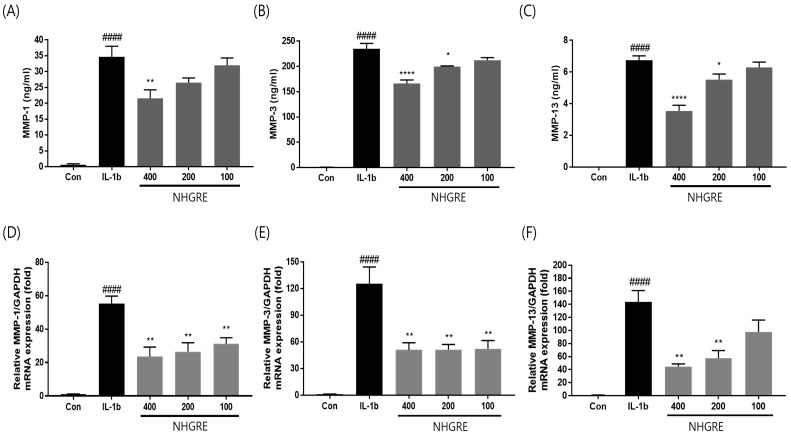
Inhibitory effects of NHGRE on matrix metalloproteinase expression in SW1353 chondrocytes treated with IL-1β. (**A**–**C**) Protein and (**D**–**F**) mRNA expression levels of MMP-1, MMP-3, and MMP-13. Each value represents mean ± SD. ^####^ *p* < 0.0001 compared to untreated control. * *p* < 0.05, ** *p* < 0.01, and **** *p* < 0.0001 compared to IL-1β-treated group. *n* = 3.

The enzyme-linked immunosorbent assay (ELISA) showed an increased production of MMP-1, -3, and -13 in the cell culture supernatants after stimulation with IL-1β; however, the pretreatment of SW1353 cells with NHGRE reduced MMP-1, -3, and -13 production in a dose-dependent manner. Additionally, the qRT-PCR results demonstrated the same trend (Figure 4D–F). These findings suggest that NHGRE decreased the production of MMPs, which could prevent ECM degradation in IL-1β-stimulated SW1353 cells.

### 2.6. Effects of NHGRE on Type II Collagen Degradation in IL-1β-Stimulated SW1353 Cells

**Figure 5:**
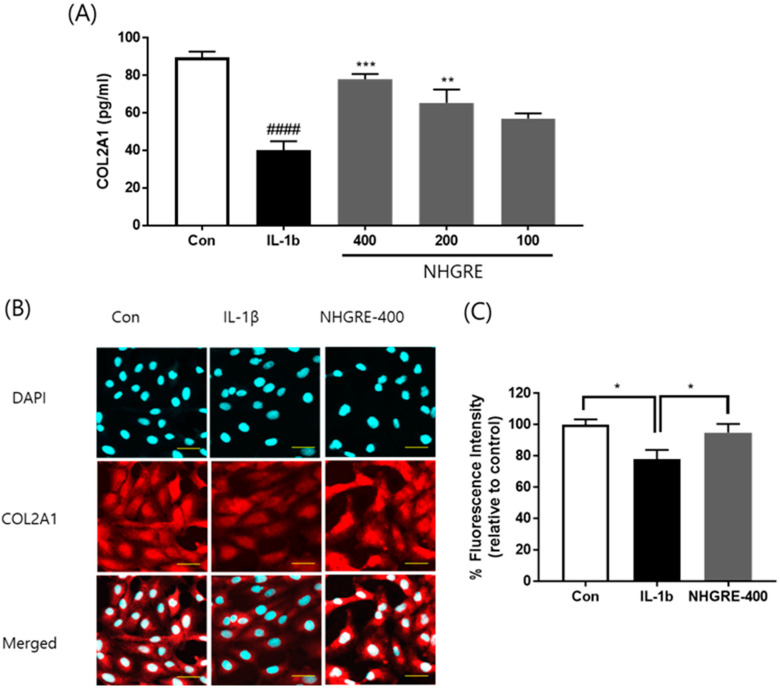
Effects of NHGRE on IL-1β-induced ECM degradation in IL-1β-treated SW1353 chondrocytes. (**A**) Protein expression, (**B**) immunofluorescence staining of type II collagen and DAPI staining of nuclei in chondrocytes, and (**C**) fluorescence intensity of type II collagen. Scale bar 20 µm. Values are expressed as mean ± SD (*n* = 3). ^####^ *p* < 0.0001 vs. untreated control. * *p* < 0.05, ** *p* < 0.01 and *** *p* < 0.001 vs. IL-1β-treated group.

As shown in Figure 5A, IL-1β treatment significantly reduced type II collagen (COL2A1) expression, and NHGRE pretreatment mitigated this effect in a dose-dependent manner. Furthermore, an immunofluorescence analysis showed that IL-1β treatment effectively decreased the expression of type II collagen (Figure 5B). In contrast, NHGRE treatment notably inhibited IL-1β-stimulated cytoplasmic type II collagen degradation.

### 2.7. Effects of NHGRE on IL-1β-Stimulated NF-κB Activation

**Figure 6:**
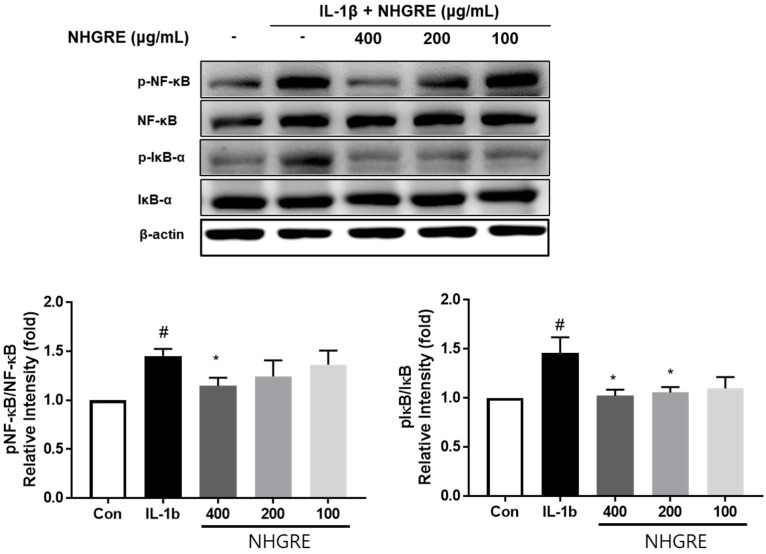
Effects of NHGRE on IL-1β-induced NF-κB activation in SW1353 chondrocytes. Protein expression and quantification of p-p65, p65, p-IκB-α, and IκB-α. Values are expressed as mean ± SD (*n* = 3). ^#^ *p* < 0.05 vs. control. * *p* < 0.05 vs. IL-1β-induced group.

To further explore the mechanism underlying the anti-inflammatory action of NHGRE, we investigated the protective effect of NHGRE on IL-1β activation in the NF-κB pathway using Western blotting. IL-1β significantly promoted the phosphorylation of p65- and IκBα, whereas NHGRE treatment showed a pronounced inhibitory effect on IL-1β-induced p65- and IκBα phosphorylation in a dose-dependent manner (Figure 6).

## 3. Discussion

Inflammation is a major factor in OA development. It is associated with the risk of the progression of cartilage destruction and exacerbates the signs and symptoms of OA such as joint pain, redness, heat, stiffness, and swelling [17]. Inflammation initiates and progresses within inflammatory cells when arachidonic acid (AA) is metabolised by COX and LOX to PGE2 and leukotriene (LT) B4, respectively [18]. Currently, treatment for the alleviation of OA symptoms includes the use of non-steroidal anti-inflammatory drugs (NSAIDs), which mainly inhibit COX expression and reduce prostaglandin (PG) synthesis [19]. The inhibition of the COX-2 enzyme pathway by NSAIDs leads to the substrate diversion of AA metabolism to the other major LOX pathway, resulting in an increased LT production and increased inflammatory reaction. Leukotriene B4 (LTB4) terminates the 5-LOX pathway. LTB4 is a mediator of inflammation in several diseases such as atherosclerosis, cancer, and cardiovascular disease [20,21]. Therefore, new anti-inflammatory agents that can simultaneously inhibit the COX-2 and 5-LOX pathways show additional anti-inflammatory effects and improved safety compared to COX inhibitors alone. In this study, the potential of NHGRE to inhibit COX-1, COX-2, and 5-LOX enzyme activities was evaluated. NHGRE showed a good inhibitory effect on the tested enzymes, with an IC_50_ = 62.00 μg/mL for COX-1, 82.33 μg/mL for COX-2, and 270.95 μg/mL for 5-LOX. The carrageenan-induced rat paw oedema model is suitable for the evaluation of the effects of anti-inflammatory agents; it has been frequently used to evaluate the anti-inflammatory efficacy of natural products [22]. In this study, NHGRE treatment at 200 mg/kg significantly inhibited paw oedema formation in rats 5 h after carrageenan injection. Additionally, the acetic acid-induced writhing model is used for screening and assessing anti-inflammatory agents or for evaluating the peripheral antinociceptive effects of drugs, because acetic acid induces abdominal contractions or writhing in mice by increasing the levels of pain mediators such as PGE2 [1,23]. In this study, the oral administration of NHGRE at all doses significantly reduced the number of writhing responses in mice compared with that in the control group. Considering that the percentage of suppression elicited by NHGRE at 150 and 200 mg/kg was similar, 150 mg/kg NHGRE was probably the maximum analgesic dose. We demonstrated that, in the MIA-induced OA rat model, NHGRE inhibits the expression of both PGE2 and LTB4, which are products of the COX-2 and 5-lipoxygenase pathways [16]. Based on the results of these in vivo studies, we propose that NHGRE exerts its inflammatory and analgesic effects through a related AA metabolism.

Inflammation is ubiquitous during the progression of OA [24]. The inhibition of the expression of pro-inflammatory cytokines and IL-6- and IL-1β-induced inflammatory mediators such as NO and PGE2 alleviate OA development and reduce inflammation, pain, and proteoglycan loss [25,26,27]. To mimic the initiation and pathogenesis of OA, in vitro investigations generally use IL-1β to induce inflammatory responses in SW1353 cells [28]. In this study, we found that IL-1β induced the production of inflammatory cytokines and mediators (TNF-α, IL-6, and PGE2) in SW1353 cells and that the expression of these inflammatory factors was suppressed by NHGRE, suggesting that NHGRE may exert an anti-inflammatory effect against IL-1β-induced inflammatory responses in SW1353.

The excessive production of inflammatory cytokines, such as TNF-α, IL-1, IL-6, and IL-8, triggers joint inflammation, which leads to ECM breakdown and cartilage degeneration [29]. Notably, IL-1β induces the release of cartilage-degrading enzymes such as MMPs and the ADAMTS family members, which consequently promotes collagen II and aggrecan degradation [30]. Furthermore, the destruction of articular cartilage in OA is due to an imbalance between the anabolic and catabolic processes in the ECM [31]. ADAMTS5, MMP1, MMP3, and MMP13 are the most commonly reported enzymes in this catabolic dysregulation, whereas type II collagen is involved in its anabolism [32]. Type II collagen is a major constituent of the cartilage tissue, and a decrease in its expression is one of the hallmarks of cartilage degeneration [33]. Therefore, MMP inhibition and type II collagen up-regulation may prevent cartilage ECM loss and cartilage degradation. In this study, IL-1β treatment notably induced the expression of MMP1, MMP3, and MMP13, which in turn reduced the type II collagen level. In contrast, NHGRE treatment reversed the MMP and type II collagen levels, suggesting that NHGRE may exert a protective effect against IL-1β-induced cartilage degeneration.

NF-κB plays a crucial role in inflammation [34]. NF-κB, when activated, inhibits type II collagen expression and increases MMP (MMP-1, -2, -3, -7, -8, -9, and -13) and aggrecanase (ADAMTS4 and ADAMTS5) levels. It also increases NO, iNOS, COX-2, and PGE-2 production, which promotes IL-1β-mediated OA progression [35,36]. Thus, NF-κB plays a decisive role in OA progress; therefore, targeting the NF-κB signalling pathway is a promising therapeutic strategy for OA [37]. The present study’s results showed that NHGRE blocks the IL-1β-mediated inflammatory response via the NF-κB signalling pathway in SW1353 cells, suggesting that the NF-κB signalling pathway may be involved in the cartilage-protective effect of NHGRE in the IL-1β-induced SW1353 cell model. Taken together, our results demonstrate that NHGRE reduces the levels of inflammation-related markers including pro-inflammatory mediators, cytokines, and enzymes by down-regulating the NF-κB signalling pathway activities. Furthermore, the inhibition of MMP expression and the up-regulation of type II collagen expression by controlling inflammation exerted chondroprotective effects by preventing cartilage ECM loss and cartilage degradation.

In our previous study, we found that NHGRE contains mogroside II, mogroside III, mogroside IV, mogroside V (MV), and 11-oxo-mogroside V [38]. The study was performed using NHGRE in which the concentration of the major component, MV, was standardised to 5.3 mg/g [16]. Although MV is known to have the highest pharmacological activity, its mechanism of action in OA is unknown, warranting further in vitro and in vivo studies.

## 4. Materials and Methods

### 4.1. Preparation of NHGRE

NHGRE was prepared according to a previously published method [16]. NHGRE was provided by Dongkuk Pharmaceutical (Seoul, Republic of Korea). It was manufactured as follows. Air-dried *S. grosvenorii* residue was extracted twice using 70% ethanol. The extract was filtered, concentrated under reduced pressure, and mixed with food-grade maltodextrin at a 1:1 ratio. The mixture was dried to obtain a powdered sample, which was then ground uniformly for use in experiments (batch No. LHGE-210417; Hunan, Huacheng Biotech Inc., Changsha, China).

### 4.2. COX and 5-LOX Activity Assay

Inhibition COX and LOX activities were determined using the COX inhibitor screening assay kit (Catalogue no. 760111; Cayman Chem., Ann Arbor, MI, USA) and 5-LOX inhibitor screening assay kit (Catalogue no. 760700; Cayman Chem.), respectively. The IC_50_ value of NHGRE was determine according to the manufacturer’s protocol.

### 4.3. Acetic Acid-Induced Writhing Response

Male ICR mice aged 7 weeks were supplied by Orient Bio (Seongnam, Republic of Korea). The mice were randomly assigned to different groups (*n* = 5). Vehicle (0.5% carboxymethyl cellulose (CMC)), NHGRE (75, 150, and 200 mg/kg), or diclofenac (10 mg/kg) was orally administered to the mice in each group. After 1 h, acetic acid (0.75%, 10 µL/g) was intraperitoneally injected into the mice. After 5 min, the number of writhes that occurred within a period of 10 min was recorded. The responses observed included stretching, the extension of the hind legs, and tension on one side [39]. All experiments were performed in accordance with the guidelines of the Korea Institute of Oriental Medicine (KIOM) (Approval Number: 22-102).

### 4.4. Carrageenan-Induced Paw Oedema

A carrageenan-induced rat hind paw oedema model was used generated using a previously described method, with some modifications [40]. Seven-week-old male ICR mice were supplied by Orient Bio. The mice were randomly assigned to different groups (*n* = 5). Vehicle (0.5% carboxymethyl cellulose (CMC)), NHGRE (150 and 200 mg/kg), or indomethacin (5 mg/kg) was orally administered to the mice in each group. After 1 h, carrageenan (1%, 50 µL) was administered to the mice via intraplantar injection. Paw oedema was measured before carrageenan injection and at 1, 3, and 5 h post-injection based on the change in paw volume (V_time_ − V_zero_). V_zero_ is the baseline paw volume for each group before carrageenan injection. V_time_ is the paw volume for each group at 1, 3, and 5 h after carrageenan injection. The percentage of paw oedema was calculated using the following equation:

Percentage of paw oedema = (V_time_ − V_zero_)/V_zero_ × 100

### 4.5. Cell Culture and Cytotoxicity Measurement

SW1353 human chondrocytes were purchased from the American Type Culture Collection (ATCC, Manassas, VA, USA). The cells were cultured in Dulbecco’s modified Eagle’s medium/nutrient mixture F-12 (DMEM/F12; Gibco-BRL, Grand Island, NY, USA), which contained 10% foetal bovine serum (FBS) and 1% penicillin/streptomycin (Gibco-Invitrogen, Carlsbad, CA, USA) under 5% CO_2_ at 37 °C. The cells were cultured in a 96-well plate and incubated for 24 h. After incubation with NHGRE at various concentrations for 2 h, the cells were stimulated with IL-1β (10 ng/mL) for 24 h. For determining cell viability, after all treatments, the medium was removed, and 3-(4,5-dimethylthiazol2-yl)-2,5-diphenyltetrazolium bromide (MTT) solution was added to each well. Following incubation in the dark for 2 h, the supernatant was replaced with an equal volume of DMSO to dissolve blue formazan crystals. The absorbance of the samples was determined at 570 nm using a microplate reader (Bio-Rad, Hercules, CA, USA) as previously described [41].

### 4.6. ELISA

The inhibitory effects of NHGRE on the expression of pro-inflammatory cytokines and mediators, namely, TNF-α, IL-6, PGE2, MMP-1, MMP-3, MMP-13, and type II collagen, were examined using commercial ELISA kits from R&D Systems (Minneapolis, MN, USA).

### 4.7. RNA Isolation and RT-PCR

Total RNA was extracted from cells using the RNeasy Mini Kit (Qiagen Inc., Valencia, CA, USA), according to the manufacturer’s instructions and quantified. cDNA synthesis was performed using the iScript cDNA Synthesis Kit (Bio-Rad, Hercules, CA, USA) and amplified with SYBR Green Supermix on a CFX Connect Real-Time PCR System (Bio-Rad, Hercules, CA, USA) under the following conditions: 10 min at 95 °C, 40 cycles at 95 °C for 15 s, 60 °C for 20 s, and 72 °C for 20 s. After determining the cycle threshold (Ct) values, we normalised the target mRNA level to the glyceraldehyde-3-phosphate dehydrogenase (*GAPDH*) level and calculated the relative mRNA levels of different target genes using the 2^−ΔΔct^ method. The primer sequences used are presented in Table 2.

### 4.8. Western Blotting

The protein was extracted from cells using RIPA buffer. Samples were electrophoresed on 10% polyacrylamide gels and transferred onto PVDF membranes. Following 1 h blocking with 5% non-fat milk, the membranes were incubated with primary antibodies (1:1000 dilution; Santa Cruz Biotechnologies, Santa Cruz, CA, USA) overnight at 4 °C. Next, the membranes were washed with TBST and incubated with horseradish peroxidase-linked secondary antibodies (diluted 1:2000) for 60 min at room temperature. Following TBST washes, the signals were detected using SuperSignal Chemiluminescence Reagent (Thermo Scientific, Atto Corporation, Tokyo, Japan) using an image analyser (LAS 4000 mini; GE Healthcare Bio-Sciences, Piscataway, NJ, USA).

### 4.9. Immunofluorescence Staining

The cells were fixed in 4% paraformaldehyde solution (pH 7.4) for 30 min at room temperature. The cells were permeabilised with 0.3% Triton X-100 in PBS for 20 min and blocked with 5% bovine serum albumin in PBS for 30 min. The cells were then incubated with anti-collagen II (1:200 dilution; Santa Cruz Biotechnologies, Santa Cruz, CA, USA) for 1 h and Texas Red-conjugated secondary antibodies (1:100 dilution) for 1 h at room temperature in the dark. After washing thrice with PBS, the cells were stained with 4–6-diamidino-2-phenylindole (DAPI) for 10 min. The slides were covered with mounting media and visualised using a Fluoview FV10i confocal microscope (Olympus, Tokyo, Japan). Fluorescence data were exported from generated images; they are expressed as the percentage of fluorescence intensity relative to that of the control. 

### 4.10. Statistical Analyses

All data are expressed as the mean ± standard deviation (SD); they were analysed using the Prism 7.0 software (GraphPad Software, Boston, MA, USA). Statistical comparisons of more than two groups were performed by the one-way analysis of variance, followed by Dunnett’s test. Results with *p* < 0.05 were considered statistically significant.

## 5. Conclusions

Collectively, the in vitro results of the present study showed that NHGRE down-regulates the IL-1β-induced expression of inflammatory mediators, including TNF-α, IL-6, and PGE2, and cartilage-degrading enzymes, such as MMP-1, MMP-3, and MMP-13. It up-regulates type II collagen levels. These results showed that NHGRE protected chondrocytes by inhibiting IL-1β-stimulated anti-inflammatory activity and cartilage degeneration by regulating the NF-κB signalling pathway. Our findings suggest the potential of NHGRE as an effective therapeutic agent for OA.

## Figures and Tables

**Table 1:** Effect of NHGRE on COX-1, COX-2, and 5-LOX enzyme activities.

Enzyme	NHGRE Dose (µg/mL)	Inhibition Rates (%)	IC_50_ (µg/mL)
COX-1	25	29.29 ± 5.21	62.00 ± 3.59
	50	44.63 ± 12.03	
	100	70.16 ± 2.96	
COX-2	25	14.02 ± 9.79	82.33 ± 9.90
	50	38.00 ± 5.52	
	100	58.56 ± 10.40	
5-LOX	100	41.37 ± 0.15	270.95 ± 6.63
	250	48.89 ± 0.73	
	500	61.62 ± 0.44	

**Table 2:** Sequences of the primers used for the real-time PCR.

Gene	Direction	Primer Sequence
*MMP-1*	Forward	5′-GACAGAGATGAAGTCCGGTTT-3′
	Reverse	5′-GCCAAAGGAGCTGTAGATGTC-3′
*MMP-3*	Forward	5′-ATTCCATGGAGCCAGGCTTTC-3′
	Reverse	5′-CATTTGGGTCAAACTCCAACTGT-3′
*MMP-13*	Forward	5′-AGCCACTTTATGCTTCCTGA-3′
	Reverse	5′-TGGCATCAAGGGATAAGGAAG-3′
*GAPDH*	Forward	5′-CACCCACTCCTCCACCTTTG-3′
	Reverse	5′-CCACCACCCTGTGCTGTAG-3′

## Data Availability

All data are available within the article.
